# Supplementary material for: The role of the γ subunit in the photosystem of the lowest-energy phototrophs
Source: Biochem J. 2022 Dec 19;479(24):2449–63. doi: 10.1042/BCJ20220508 (PMC9788563; doi:10.1042/BCJ20220508)
Supplement: Supplementary Material [file BCJ-479-2449-s1.pdf]

SUPPLEMENTARY TABLES & FIGURES

Strain/Plasmid	Properties	Source
<u>*E. coli*</u>		
JM109	Cloning strain for pK18*mobsacB* and pBBRBB constructs	Promega
ST18 (DSM 22074)	Conjugative strain for pK18*mobsacB* and pBBRBB constructs	DSMZ
<u>*Blc. viridis*</u>		
WT	DSM-133	DSMZ
$\Delta LH1\gamma_{1-3}$	Replacement of BVIR_1786–1788 with *aadA* from pSRA81 in WT, Sp^R	This study
$\Delta LH1\gamma_{1-4}$	Replacement of BVIR_2981 with *bla* from pET-3a in $\Delta LH1\gamma_{1-3}$, Sp^R Amp^R	This study
$\Delta LH1\gamma_{1-4}$ + $LH1\gamma_1$	$\Delta LH1\gamma_{1-4}$ harbouring pBBRBB-Ppuf^{Bv}[$LH1\gamma_1$], Sp^R Amp^R Km^R	This study
$\Delta LH1\gamma_{1-4}$ + $LH1\gamma_4$	$\Delta LH1\gamma_{1-4}$ harbouring pBBRBB-Ppuf^{Bv}[$LH1\gamma_4$], Sp^R Amp^R Km^R	This study
<u>Plasmid</u>		
pK18*mobsacB*	Allelic exchange vector, Km^R	J. Armitage*, [2]
pBBRBB-P$puf_{843\text{-}1200}$-DsRed	Purple bacterial expression vector carrying the *Rhodobacter sphaeroides puf* promoter, Km^R	Addgene, [3]
pBBRBB-Ppuf^{Bv}	Replacement of promoter in pBBRBB-P$puf_{843\text{-}1200}$-DsRed with equivalent from *Blc. viridis*, Km^R	This study
pBBRBB-Ppuf^{Bv}[$LH1\gamma_1$]	BVIR_1786 cloned downstream of promoter in pBBRBB-Ppuf^{Bv}	This study
pBBRBB-Ppuf^{Bv}[$LH1\gamma_4$]	BVIR_2981 cloned downstream of promoter in pBBRBB-Ppuf^{Bv}	This study
pSRA81	Source of *aadA* cassette, Sp^R	[4]
pET-3a	Source of *bla* cassette, Amp^R	Novagen

Supplementary Table 1. List of strains and plasmids described in this study

* Department of Biochemistry, University of Oxford, South Parks Road, Oxford OX1 3QU, U.K.

[1] Simon R, Priefer U, Pühler A (1983) A broad host range mobilization system for *in vivo* genetic engineering: transposon mutagenesis in Gram negative bacteria. *Nat Biotechnol* **1**:784–791

[2] Schäfer A, Tauch A, Jäger W, Kalinowski J, Thierbach G, Pühler A. (1994). Small mobilizable multi-purpose cloning vectors derived from the Escherichia coli plasmids pK18 and pK19: selection of defined deletions in the chromosome of Corynebacterium glutamicum. *Gene* 145:69–73

[3] Tikh IB, Held M, Schmidt-Dannert C. (2014) BioBrick™ compatible vector system for protein expression in *Rhodobacter sphaeroides*. *Appl Microbiol Biotechnol* **98**:3111–3119

[4] Canniffe DP, Thweatt JL, Chew AG, Hunter CN, Bryant DA. (2018) A paralog of a bacteriochlorophyll biosynthesis enzyme catalyzes the formation of 1, 2-dihydrocarotenoids in green sulfur bacteria. *J Biol Chem* **293**:15233–15242

Primer	Sequence (5’-3’)	Cleavage site
LH1γ_{1-3}UpF	CCGGAATTCCCTTGAACCAGGCCTCCTCGCC	EcoRI
LH1γ_{1-3}UpR	CCAAAAAAACAGTCATAACAAGCCATCGTTGGTCCTCTCATGACGGGTC	
LH1γ_{1-3}DownF	CACCAAGGTAGTCGGCAAATAAGAATTGTCGGGTCCGGCCCCTATCG	
LH1γ_{1-3}DownR	CCCAAGCTTGGTGTTTGCCACCGCCATCGTCCTTG	HindIII
aadAF	ATGGCTTGTTATGACTGTTTTTTTGG	
aadAR	TTATTTGCCGACTACCTTGGTG	
LH1γ_{1-3}CheckF	CCAGGGCGTAATCCTCGGTGTC	
LH1γ_{1-3}CheckR	GCCAAGCTCCGCACCACGG	
LH1γ_4UpF	GAGTCTAGACCTTGGCTCCACCAAATTTCTTTTGCC	XbaI
LH1γ_4UpR	CGCACATTTCCCCGAAAAGTGCCGCTTTCTTCATTTTGCAGACTCC	
LH1γ_4DownF	CCTCACTGATTAAGCATTGGTAACTGCTAGTGACACGGTTTCCGGCC	
LH1γ_4DownR	CCCAAGCTTCTACGACCAGATCGCGGTCTCC	HindIII
blaF	GGCACTTTTCGGGGAAATGTGCG	
blaR	CAGTTACCAATGCTTAATCAGTGAGG	
LH1γ_4CheckF	GAATGGCATTCAAGAGGTCAGG	
LH1γ_4CheckR	GGCTTCCACTTCATGAACAAGG	
Ppuf^{Bv}F	GCTCTAGAGCTGATCCTCGACCATGATCG	XbaI
Ppuf^{Bv}R	CGAGATCTACCCTCATCAATGCGGGCC	BglII
LH1γ_1BBF	GCAGATCTATGAAACTTTCAGCTATTCTTG	BglII
LH1γ_1BBR	CTGACTAGTTCAACGATAGGTCAGCGCAATC	SpeI
LH1γ_4BBF	GCAGATCTATGAAGAAAGCATCTGCAATC	BglII
LH1γ_4BBR	CTGACTAGTCTAGTTGTAAACGAAGGCAATC	SpeI

Supplementary Table 2. List of primers used in this study

Restriction enzyme cleavage sites used for cloning are underlined in the primer sequence.

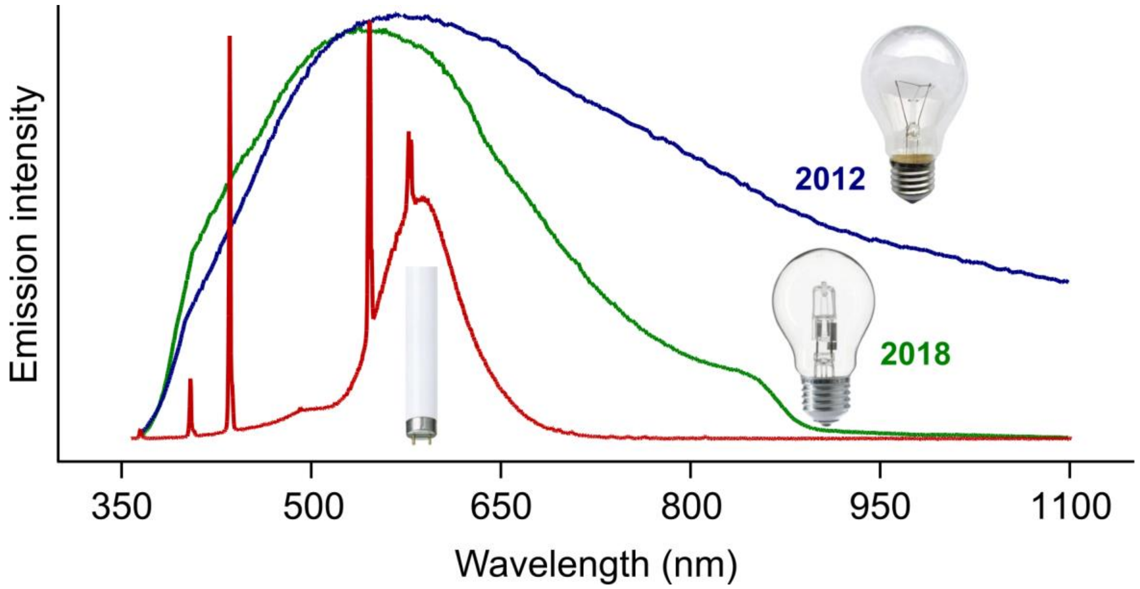

Figure S1. Emission spectra of bulbs used for growth of *Blc. viridis* strains. The year in which production of incandescent (blue) and halogen (green) bulbs were banned in the EU are labelled.

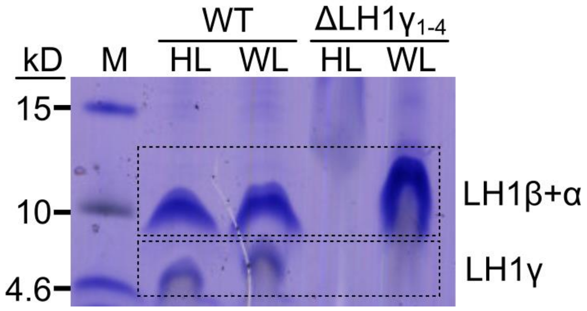

Figure S2. Electrophoretic separation of LH1 components on a TRIS-tricine gel.

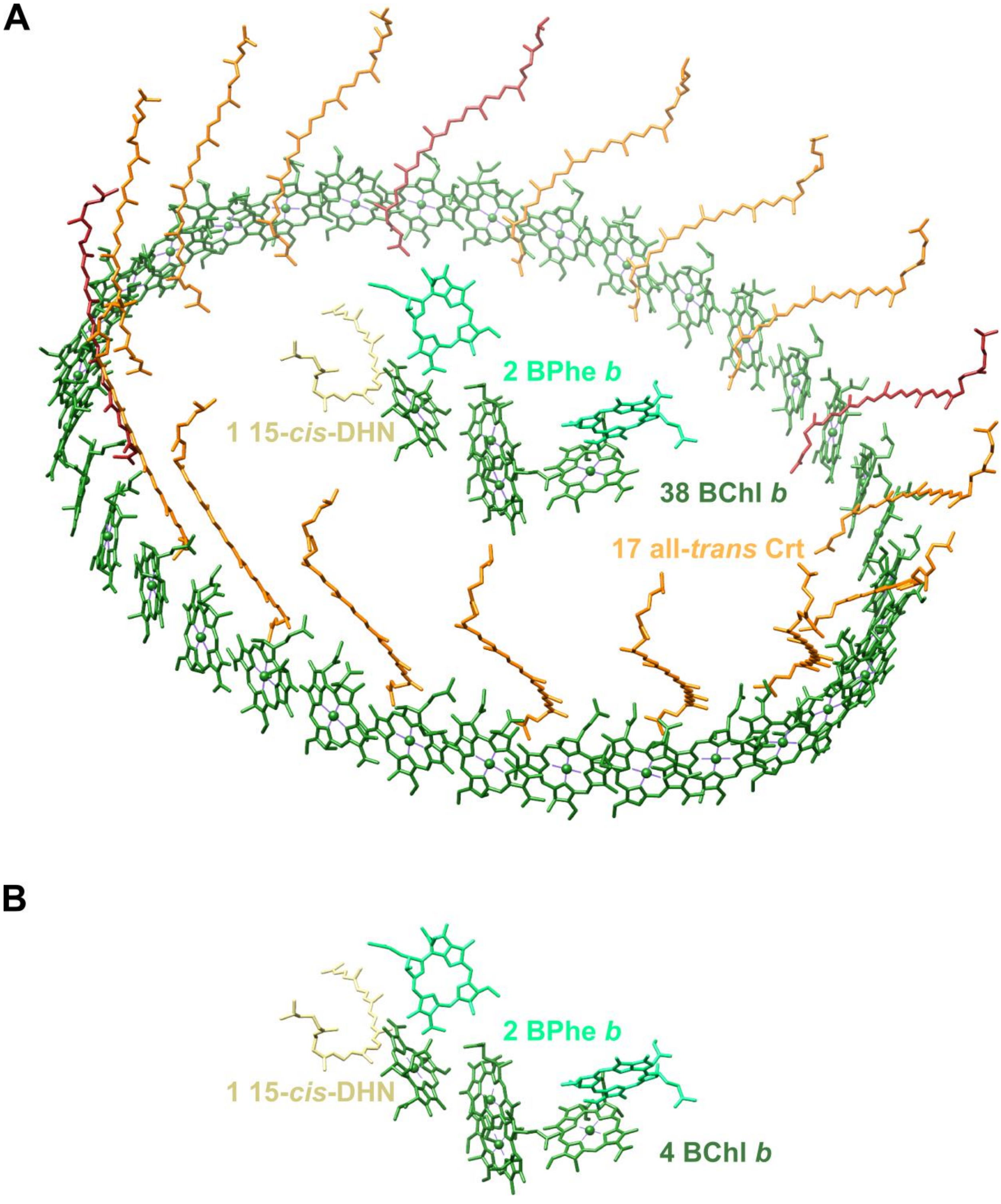

Figure S3. Arrangement and number of pigments in A) RC–LH1 and B) RC of *Blc. viridis*. Phytyl tails have been removed from BChls and BPhes for clarity. The 17 all-trans carotenoids in LH1 are coloured in orange (neurosporene species) or red (lycopene species) according to their approximate abundance in WT cells.
